# Structure–Activity Relationship and Molecular Docking of Natural Product Library Reveal Chrysin as a Novel Dipeptidyl Peptidase-4 (DPP-4) Inhibitor: An Integrated In Silico and In Vitro Study

**DOI:** 10.3390/molecules23061368

**Published:** 2018-06-06

**Authors:** Poonam Kalhotra, Veera C. S. R. Chittepu, Guillermo Osorio-Revilla, Tzayhri Gallardo-Velázquez

**Affiliations:** 1 Departamento de Biofísica, Escuela Nacional de Ciencias Biológicas, Instituto Politécnico Nacional, Prolongación de Carpio y Plan de Ayala S/N. Col. Santo Tomás, C.P. 11340 Ciudad de México, Mexico; kalhotrapoonam@gmail.com; 2 Departamento de Ingeniería Bioquímica, Escuela Nacional de Ciencias Biológicas, Instituto Politécnico Nacional, Av. Wilfrido Massieu S/N, Col. Unidad Profesional Adolfo López Mateos, Zacatenco, C.P. 07738 Ciudad de México, Mexico; veerareddy9@gmail.com (V.C.S.R.C.); osorgi@gmail.com (G.O.-R.)

**Keywords:** natural products, field-based virtual screening, structure–activity relationship model, molecular docking, dipeptidyl peptidase-4 enzyme

## Abstract

Numerous studies indicate that diets with a variety of fruits and vegetables decrease the incidence of severe diseases, like diabetes, obesity, and cancer. Diets contain a variety of bioactive compounds, and their features, like diverge scaffolds, and structural complexity make them the most successful source of potential leads or hits in the process of drug discovery and drug development. Recently, novel serine protease dipeptidyl peptidase-4 (DPP-4) inhibitors played a role in the management of diabetes, obesity, and cancer. This study describes the development of field template, field-based qualitative structure–activity relationship (SAR) model demonstrating DPP-4 inhibitors of natural origin, and the same model is used to screen virtually focused food database composed of polyphenols as potential DPP-4 inhibitors. Compounds’ similarity to field template, and novelty score “high and very high”, were used as primary criteria to identify novel DPP-4 inhibitors. Molecular docking simulations were performed on the resulting natural compounds using FlexX algorithm. Finally, one natural compound, chrysin, was chosen to be evaluated experimentally to demonstrate the applicability of constructed SAR model. This study provides the molecular insights necessary in the discovery of new leads as DPP-4 inhibitors, to improve the potency of existing DPP-4 natural inhibitors.

## 1. Introduction

Plant-based systems are proven to be rich sources of bioactive compounds, which were used by different ancient cultures primarily in North America, and plants remained as source of new leads and new chemicals entities in drug discovery [1,2,3]. Galanthamine HBr, miglustat, DMXAA, and delta-9-THC are some of the plant derived compounds approved as therapeutics from projects related to drug discovery and drug development t [4,5].

Diet has played a role in the prevention, control, and treatment of diseases like diabetes [6], obesity [7], and cancer [8]. These are serious health problems faced by individuals in both developed and developing countries. Intestinal related L1-cells and duodenal related K-cells release incretin hormones named glucagon-like peptide-1 (GLP-1), and glucose-dependent insulinotropic polypeptide (GIP), respectively, in response to food intake. The GLP-1 incretin hormone stimulates insulin secretion, decreases glucagon release, increases beta-cell mass, and decreases beta-cell apoptosis, thereby helping to regulate glucose metabolism. These hormones were rapidly degraded and inactivated by the activity of an enzyme called dipeptidyl peptidase (DPP-4) [9]. Inhibition of the DPP-4 enzyme played an important role in controlling the phenotypes of severe diseases, such as diabetes, obesity, and cancer, by preventing the inhibition of incretin hormones [10,11,12].

Based on the literature study, there are currently 77 entries for human DPP-4-inhibitor complexes. Analyzing DPP-4 crystal structures and understanding of their SAR revealed essential regions considered to be crucial for the inhibition of DPP-4 activity. The critical regions and residues responsible for DPP-4 inhibition are (a) hydrophobic S1 pocket residues Tyr 631, Val 656, Trp 659, Tyr 662, Tyr 666, and Val 711, (b) N-terminal recognition region residues Glu 205, and Glu 206, (c) enzyme catalytic residues Ser 630, Asp 708, and His 740, and (d) hydrophobic S2 pocket residues Arg 125, Phe 357, Arg 358, Tyr 547, Pro 550, and Asn 710 [13,14,15]. Extensive research on DPP-4 enzyme had resulted in the development of sitagliptin, vildagliptin, saxagliptin, and alogliptin, which are some of the approved DPP-4 inhibitors in clinical use. However, they possess side effects like pancreatitis, cardiovascular problems, renal toxicity, hepatic toxicity, and are precancerous [16]. As a result, there is a need to develop new compounds as DPP-4 inhibitors with fewer side effects, and natural compound identification would be a satisfactory strategy.

Recently, computational approaches had proven to be successful in identifying new potential drug targets, discovering new drugs, and developing them to succeed in the therapeutic development process at the academic and pharmaceutical industry level [17]. Computational approaches, like ligand-based virtual screening [18], structure-based virtual screening methods, like molecular docking [19], QM/MM, molecular dynamics, and integrated approaches, were proven to be successful in drug discovery and drug development [20]. Many investigations were carried out on structural characterization of natural compounds using X-ray, NMR, and other high-throughput technologies, and all the data is available in the form of databases, like Phenol-Explorer, Prime, and others [21,22]. These databases play a crucial role in performing structure-based virtual screening and ligand-based virtual screening, which are proven to be successful approaches in the process of computational drug discovery and drug development [22,23]. The field template would assist in discovering new compounds to modulate DPP-4, as well as in understanding the structure–activity relationship of these compounds [24,25]. Integration of field template and structure-based virtual screening would be beneficial for identifying new compounds to modulate drug targets [26]. Based on the literature survey, no study was carried out to utilize the field template, the field point based on virtual-screening methodologies as tools in the process of hit or lead identification, or in the lead optimization process. Additionally, their application had not yet been explored in the field of natural compounds.

Therefore, the current study is focused on the use of field-based virtual screening, and structure–activity relationship models utilizing field points to elucidate molecular insights into DPP-4 inhibitors from natural origin. The generated field template and the activity atlas model were used to screen natural compounds from the Phenol-Explorer database. Furthermore, the study of protein–ligand interactions of selected polyphenols using molecular docking had revealed the novel inhibitor of DPP-4. The hits of the DPP-4 enzyme were identified on the basis of similarity to the field template model, the novelty score, the docking score, and the interacting residues of compounds. Lastly, one of the best hits was validated experimentally to demonstrate the reliability of the generated model.

## 2. Results and Discussion

### 2.1. Field-Based Virtual Screening

#### 2.1.1. Field Template Generation

**Figure 1:**
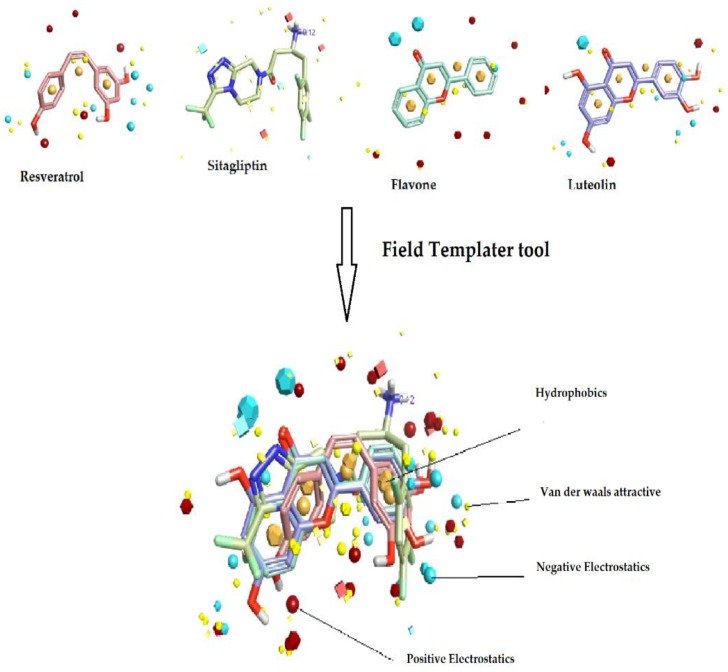
Representation of four template compounds inhibiting DPP-4 activity and identification of three-dimensional bioactive conformations on the basis of field points generated using field template tool.

In this study, four diverse compounds named resveratrol, sitagliptin, flavone, and luteolin already proven to inhibit DPP-4 activity, were retrieved and used to generate three dimensional (3D) bioactive conformations and pharmacophore hypothesis (also called the field template). The generated hypothesis is represented as a function of electrostatic and hydrophobic fields [27], and the created field template is shown in Figure 1. The field template is visualized using Forge Visualization, and it represents the consensus alignment and bioactive conformation of the four compounds. The field template tool had resulted in 419 pharmacophore hypotheses. The top scoring template showed 59.3% general similarity, 50.5% field similarity, and 68% shape similarity, which were chosen as the best field template. The resulting template was used as a reference template in the process of building the structure–activity relationship (SAR) model using the Activity Atlas module.

#### 2.1.2. Activity Atlas Model Generation

**Figure 2:**
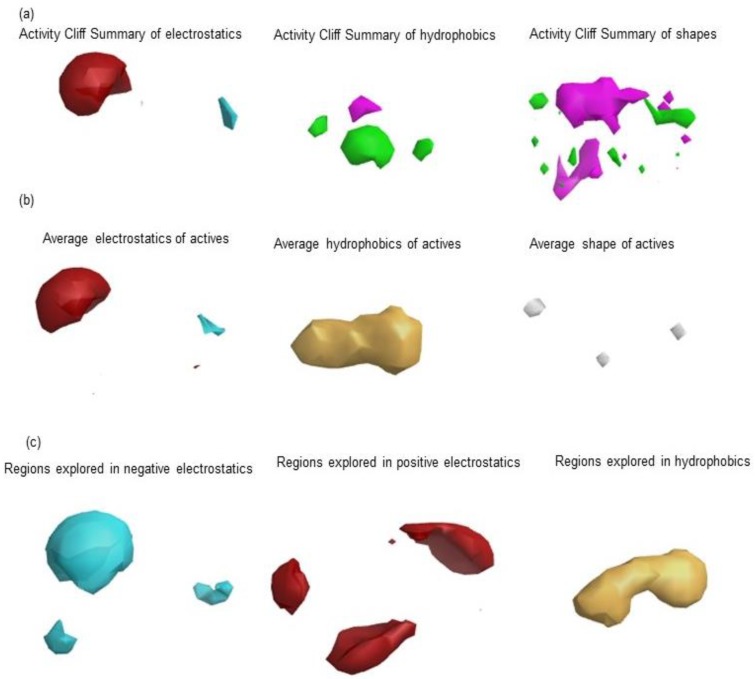
Activity atlas model generated based on the alignment of 13 polyphenols to generated field template which provides: activity cliff summary (**a**); average of actives (**b**); and regions explored (**c**). Forge visualization is used to understand the activity atlas and structure–activity relationship (SAR) of different natural compounds already proven to inhibit dipeptidyl peptidase-4 activity.

To understand the key features of DPP-4 inhibition of natural compounds for further optimization of their potency, and to help identify new natural inhibitors of DPP-4, SAR was performed by using the activity atlas model. To achieve these, the average of actives, regions of activity, and activity cliff summary were explored for the DPP-4 inhibitors of natural origin. To build the activity atlas model, a total of thirteen polyphenols (shown in Table 1), together with their inhibitory IC50 values, were collected from the literature and aligned with the generated field template [27]. The activity atlas model results are shown in Figure 2, and describes the structure–activity relationship of natural compounds inhibiting DPP-4. The generated SAR model provides information about the average of actives (see Figure 2a), activity cliff summary (see Figure 2b), and regions explored (see Figure 2c). The generated SAR model was used to screen natural product databases, like Phenol-Explorer, and assign novelty scores to each polyphenol curated in the database to identify new and novel natural compounds. The results showed that positive field regions within the SAR model, as indicated by red color sites, regulated the inhibitory activity of DPP-4. Higher field regions are associated with the higher inhibitory activity.

#### 2.1.3. Screening of Molecules Based on Field Template Alignment and Novelty Score

To identify the potential hits, polyphenols from food were retrieved from the Phenol-Explorer (version 3.6, Clermont-Ferrand, France) database (http://phenol-explorer.eu/). This database is well-characterized for retrieving information about natural compounds, especially polyphenols from food. A total of 501 polyphenols are available in this database, and these were aligned to generate the field template and the activity atlas model. The same model was utilized to score all the polyphenols with a novelty score (low, medium, high, and very high). It resulted in 153 compounds with a “high or very high” novelty scores (Supplementary Material), which were considered for the molecular docking study to understand their binding site and interacting residues responsible for inhibiting the DPP-4 enzyme.

### 2.2. Molecular Docking

**Figure 3:**
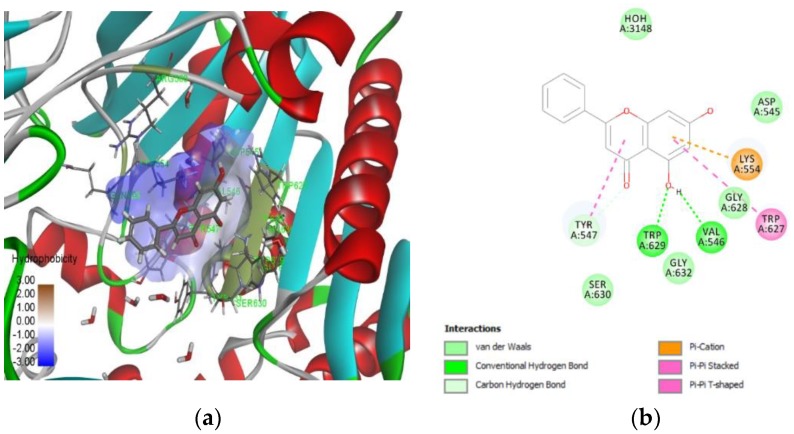
The three-dimensional view, representing the binding pose view of chrysin: at the active site of the DPP-4 enzyme (**a**); and (**b**) a two-dimensional representation of interacting residues from the DPP-4 enzyme, involved at binding pose of chrysin.

**Figure 4:**
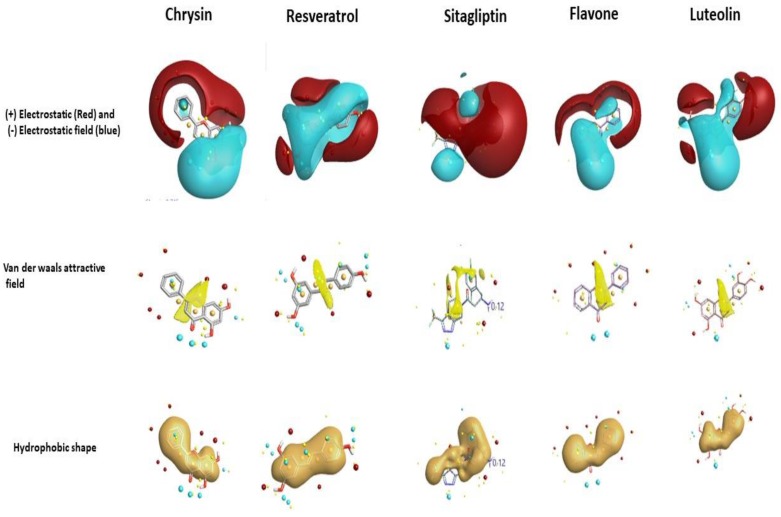
Chrysin bioactive conformation predicted using field template and fields predicted using activity atlas model. Field of chrysin is compared with resveratrol, sitagliptin, flavone, and luteolin.

In this study, the molecular docking tool FlexX provided by LeadIT software was utilized. Initially, our docking protocol was validated using redocking methodology. To achieve this task, the X-ray crystal structure related to DPP-4 in complex with vildagliptin was retrieved from the Protein Data Bank (PDB ID: 6B1E). The docking tool was able to replicate the experimental binding, and interacting residues with root mean squared deviation (RMSD) of 1.43 A^0^. A total of 153 compounds were docked to the active site of DPP-4 using the FlexX tool. Among the top scoring compounds, interacting residues involved in the binding site of the enzyme, and manually comparing the resulting natural compounds with the generated field template (shown in Supplementary Material, Table S1), the compounds with an increase in field regions were considered as hits, and among these, chrysin was chosen as a natural compound to inhibit the DPP-4 enzyme and as a chemical to be evaluated experimentally to demonstrate the applicability of the generated SAR model. Chrysin was chosen because molecular docking had revealed chrysin possesses a FlexX score of −21.4 KJ/mol and the top ranking binding pose is visualized using the Discovery studio visualizer, which is shown in Figure 3. The interacting residues involved in the binding site of DPP-4 with chrysin were TRP 629, Val 546, TRP627, LYS 554, TYR 547, SER 630, and GLY 628. It is apparent that chrysin binds to the residues, belonging to the catalytic triad (SER 630), hydrophobic S1 pocket (TYR 547). These residues belong to the DPP-4 druggable region, and chrysin possesses drug-like physicochemical properties. Figure 4 describes the activity atlas model results, which shows that chrysin possesses a similarity of 74.5% to the generated field template and the resulted fields was compared to resveratrol, sitagliptin, luteolin and flavone. The activity atlas model (SAR) revealed the novelty score of chrysin as “very high,” where a very high novelty score represents the chrysin is very different to 13 polyphenols chosen in the training dataset.

### 2.3. In Vitro Assay to Validate Chrysin as Dipeptidyl Peptidase-4 Inhibitor

**Figure 5:**
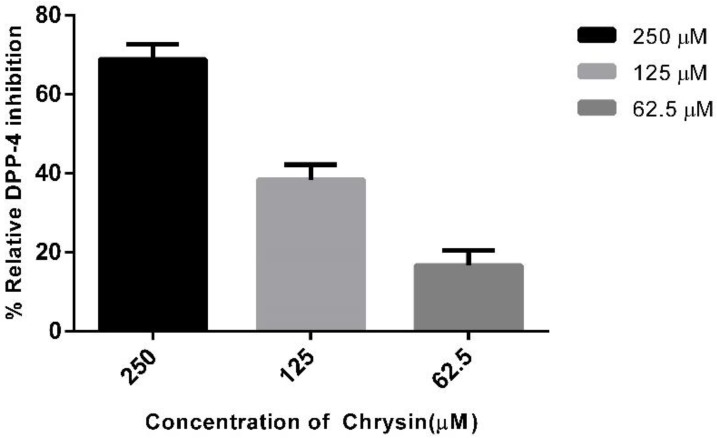
Different concentrations of chrysin and their relative percent of inhibition of DPP-4 enzyme activity. Data are represented as mean ± standard error.

Chrysin role as DPP-4 inhibitor was tested at different concentrations of 250 µM, 125 µM, and 62.5 µM, and it is observed that chrysin showed an inhibitory effect. Chrysin inhibits DPP-4 activity in a concentration-dependent manner, and the relative percentage of inhibition was 68.9%, 38.4%, and 16.6% for 250 µM, 125 µM, and 62.5 µM, respectively, which is shown in Figure 5. The observed results are in agreement with the Chinese patent (CN107496409A). Recently, a study demonstrated the role of chrysin as an antidiabetic, antidyslipidemic, and anti-inflammatory compound [28].

### 2.4. In Silico Absorption, Distribution, Metabolism, and Toxicity of Chrysin

Chrysin possesses a high gastrointestinal absorption. Also, it is permeable to the blood–brain barrier. It is not the substrate of P-glycoprotein, but it acts as a substrate to a certain cytochrome family of enzymes, like the metabolism of xenobiotic enzymes in the body cytochrome P450 1A2 (CYP1A2), cytochrome P450 2D6 (CYP2D6), and cytochrome P450 3A4 (CYP3A4, the function is to oxidize the xenobiotics). The skin permeation of chrysin is −5.35 cm/s. These results are supported by the metabolism study of chrysin in human volunteers, where it is observed that chrysin has low bioavailability, rapid hydrolysis, and fecal elimination [29,30]. In silico toxicity results revealed that there is no AMES (Salmonella typhimurium reverse mutation assay) toxicity and no carcinogenesis. In silico results revealed that chrysin has an LD50 (mol/kg) of 2.6983 mg, pLC50 (mg/L) of 0.8 mg, and pIGC50 (µg/L) of 0.334 mg.

### 2.5. Metabolic Transformation of Chrysin

**Figure 6:**
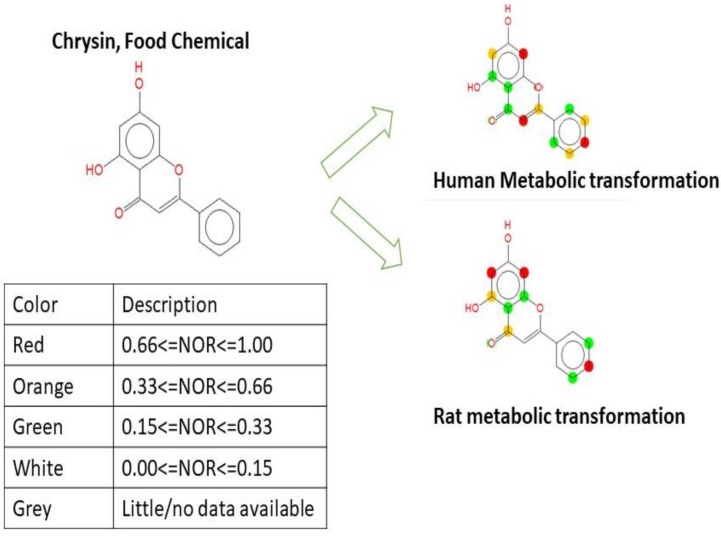
Metabolic transformation of natural compound chrysin.

To gain insight into the rapid metabolism of chrysin, a computational study on the chemical structure and metabolic transformation of chrysin was performed using MetaPrint2D, and the results are shown in Figure 6. MetaPrint2D predictions revealed that chrysin could undergo metabolization. The red color denotes a good metabolism site; the orange color denotes a moderate metabolism site; the green color denotes it is less likely to undergo metabolism; no color denotes the low probability of metabolism.

## 3. Materials and Methods

### 3.1. Generation of Field Template Hypothesis and Activity Atlas Model

As no structural information is currently available for DPP-4 inhibitors of natural origin in its protein–ligand active state, the field template tool, provided by Forge software (Cresset Inc., Cambridgeshire, UK) was used to determine the common patterns, bioactive conformations, and to perform the consensus alignments among diverse molecules. To achieve this, the field and shape information were used to create the bioactive conformations of four divergent compounds. From past studies, the bioactive compounds flavone, luteolin, resveratrol, and sitagliptin were chosen in this study because of their DPP-4 inhibition activity and the field templater tool was used to generate three dimensional (3D) bioactive conformation, field-based template (hypothesis). The developed field-based template (pharmacophore hypothesis) represents four different fields, such as positive and negative electrostatic, van der Waals shape, and hydrophobic shapes and sizes, which are responsible for inhibition of DPP-4 activity. One condition used to create the field-based template was a minimum shape similarity of 0.5. The top ranking template was chosen as the reference “field template,” to generate the activity atlas model.

The activity atlas model, a technique provided by Forge software, was utilized to generate a structure–activity relationship (SAR) model among the set of aligned biological active compounds by using the field template developed as a reference. The generated SAR model is represented as a function of their electrostatic and shape properties. The generated model can help to identify common patterns among the aligned active molecules, and common features (positive and negative electrostatics, favorable and unfavorable hydrophobic, and shape of actives). The model also helps to calculate the novelty score for the new compound of interest. The activity atlas model uses the Bayesian approach to build the SAR model. The Forge visualization tool was used to visualize and understand the key features responsible for the DPP-4 inhibition activity.

### 3.2. Field-Based and Activity Atlas Model-Based Virtual Screening

To identify the new and novel DPP-4 inhibitors, initially- the field point-based, and the activity atlas model-based virtual screening were performed on Phenol-Explorer database. Phenol-Explorer database is a focused food database that contains all polyphenols characterized so far. Based on the Tanimoto score, which assessed whether compounds had greater than 40% similarity to the generated field template, we score all the polyphenols in the database as “low, high, and very high” using Forge software (Cresset Inc., Cambridgeshire, UK).

### 3.3. Molecular Docking

The protein DPP-4 in complex with vildagliptin (PDB ID: 6B1E) was validated using the structure validation server, SAVES, and then the protein was loaded into the protein preparation module of the Biosolve IT software (GmbH, St Augustin, Germany). Chain A was selected for preparation to perform molecular docking simulation. The binding site is comprised of the binding pocket where the reference ligand vildagliptin is bound to the active site DPP-4. The energy of this site was minimized. The atomic coordinates of the binding site are converged, and the resulting protein was used for docking.

In this study, molecular docking procedure helps to predict the binding pose of natural compounds to a DPP-4 protein of interest, to calculate their binding affinity, and interacting residues involved at the binding site. In this study, the FlexX algorithm (LeadIT software package version 2.3.2 (BioSolveIT GmbH, St Augustin, Germany)) was used to score, rank, and generate the binding poses of compounds interacting with the DPP-4 protein. FlexX uses the robust incremental construction algorithm. All the docking poses and interacting studies are visualized using the Discovery Studio Visualizer 2.5.5.

### 3.4. Chemicals and Reagents

The natural compound chrysin, dipeptidyl peptidase-4 enzyme inhibitory screening kit, and dimethyl sulfoxide were purchased from Sigma Aldrich (St. Louis, MO, USA).

### 3.5. In Vitro DPP-4 Assay

The DPP-4 Inhibitor Screening Kit (Sigma Aldrich, St. Louis, MO, USA) was utilized to validate the natural compounds inhibitory effect in-vitro. Cleavage of the fluorescent product (λex = 360 nm and λem = 460 nm), proportional to enzyme activity present in a well, was monitored using a microwell plate reader (FlexStation 3 Multi-Mode Microplate Reader, Molecular Devices, LLC, San Jose, CA, USA). Fluorescence emissions were collected in kinetic mode on black well clear bottomed 96-well plates, for a duration of 30 min. The percent of relative inhibition was calculated using Formula (1), where Δ*F/*Δ*T* denotes the change in fluorescence over the chosen time interval. All the experiments were carried out in duplicates and graphs were plotted using Google Sheets.

(1) $$\%\;Relative\;inhibition=\;\frac{\left(\frac{\Delta F}{\Delta T}\right)enzyme\;-\left(\frac{\Delta F}{\Delta T}\right)enzyme\;substrate\;inhibitor}{\left(\frac{\Delta F}{\Delta T}\right)enzyme}\;\times 100$$

### 3.6. In Silico Absorption, Distribution, Metabolism, and Toxicity

In silico absorption, distribution, and metabolism were calculated using the web server SWISS ADME (http://www.swissadme.ch/index.php) [31] and toxicity parameters like AMES, carcinogenesis, rat acute toxicity LD50 (mol/kg), fish toxicity pLC50 (mg/L), and *Tetrahymena pyriformis* Toxicity pIGC50 (µg/L) were calculated using server admetSAR (http://lmmd.ecust.edu.cn:8000/) [32].

### 3.7. Metabolic Biotransformation of Chrysin

The metabolic biotransformations of the natural compound chrysin in rats, and in humans, were calculated using the MetaPrint2D module, from BioClipse [22,33].

## 4. Conclusions

A field-based qualitative structure activity relationship model was proposed as a virtual screening tool for the identification of natural compounds as DPP-4 inhibitors. This model defines the molecular features of DPP-4 inhibitors of natural origin. The key features of this model are average shape, hydrophobic regions, and positive and negative electrostatics. All polyphenols in the Phenol-Explorer database were virtually screened with the help of the derived QSAR model to identify potential hits. After the virtual screening, all the polyphenols were docked to understand the interacting residues with the DPP-4 active site. Based on the field template similarity, an increase in field regions, the molecular docking score, and the interacting residues, chrysin was discovered as a new natural compound to inhibit DPP-4. An in vitro assay revealed that chrysin inhibits DPP-4 enzyme in a concentration-dependent manner. These results may be of great help in early drug discovery, and the drug development stage. The proposed SAR model can assist in the discovery of new compounds as DPP-4 inhibitors and improve the potency of chrysin during the lead optimization process in drug discovery. Based on the results of this work, chrysin as a DPP-4 inhibitor can be used alone or in combination with current chemotherapies to improve treatment for diabetes, obesity, and cancer.

## Figures and Tables

**Table 1:** Natural products proven to inhibit dipeptidyl peptidase-4 enzyme activity.

Compound Name	Inhibitory Concentration (μM)
Hispidulin	0.49 ± 0.1
Crisimaritin	0.43 ± 0.07
Luteolin	0.12 ± 0.01
Apigenin	0.14 ± 0.02
Kaempferol	0.49 ± 0.1
Flavone	0.17 ± 0.01
Hesperetin	0.28 ± 0.07
Naringenin	2.5 ± 0.3
Genistein	0.48 ± 0.04
Cyanidin	1.41 ± 0.25
Cyanidin-3-glucoside	0.42 ± 0.09
Malvidin	1.41 ± 0.44
Resveratrol	0.0006 ± 0.0004

## Supplementary Material

The following is available online. Table S1. Top scoring compounds resulted after performing molecular docking simulations, manual comparison of their field’s positive electrostatic, negative electrostatic, hydrophobic shape, novelty score, percent of similarity to field template and natural compound interacting residues at the binding site of DPP-4 and FlexX score.
