# Supplementary material for: Structure–Activity Relationship and Molecular Docking of Natural Product Library Reveal Chrysin as a Novel Dipeptidyl Peptidase-4 (DPP-4) Inhibitor: An Integrated In Silico and In Vitro Study
Source: Molecules. 2018 Jun 6;23(6):1368. doi: 10.3390/molecules23061368 (PMC6100528; doi:10.3390/molecules23061368)
Supplement: Supplementary file 1 [file molecules-23-01368-s001.pdf]

Table S1. Resulted top 25 scoring natural compounds after Molecular docking simulations, novelty score, similarity to field template, positive electrostatics, negative electrostatics, hydrophobic shape, interacting residues at the binding site of enzyme and docking score. Color codes for interacting residues row are: red color denotes unfavorable interactions (bumps, negative-negative, donor-donor, acceptor-acceptor region, orange color indicates the formation of the salt bridge, green color denotes conventional hydrogen bond, pink color denotes pi-pi stack interactions.

Sl.No	Compound Name	Novelty Score and similarity	Positive electrostatics	Negative electrostatics	Hydrophobic Shape	Interacting Residues	Docking Score
1	Quercetin 3-O-glucuronide	Very High, 60.6	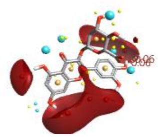	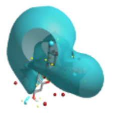	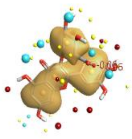	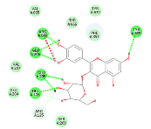	-36.253
2	3-Hydroxyhippuric acid	Very High, 61.7	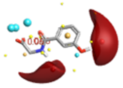	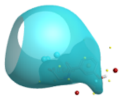	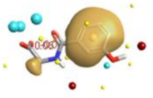	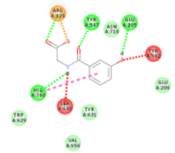	-28.003
3	Puerarin	Very High, 58.4	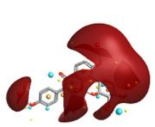	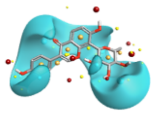	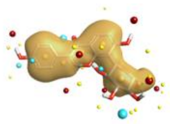	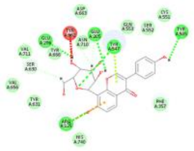	-25.875
4	trans-Resveratrol 3-sulfate	Vert High, 63.8	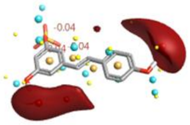	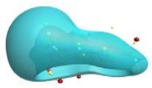	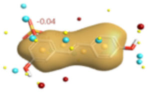	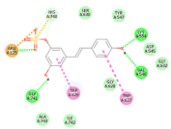	-25.691
5	Tectorigenin 4'-sulfate	Very High, 59.9	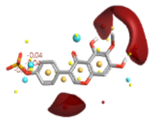	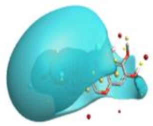	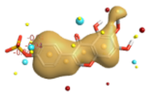	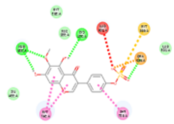	-24.937
6	Feruloylglycine	Very High, 64.4	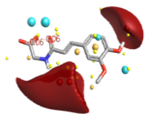	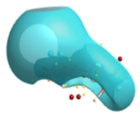	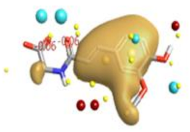	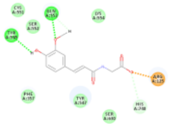	-24.833
7	cis-Resveratrol 3-sulfate	Very High, 64.0	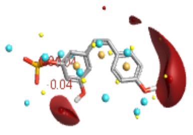	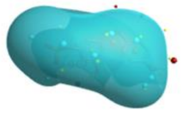	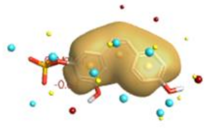	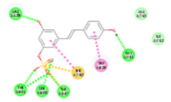	-24.621
8	Quercetin 3'-sulfate	Very High, 71.9	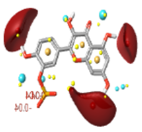	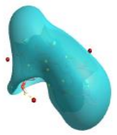	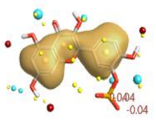	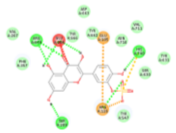	-24.502
9	Dihydroquercetin	Very High, 71.7	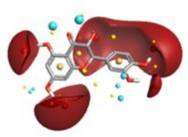	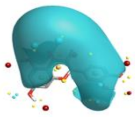	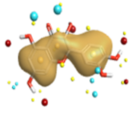	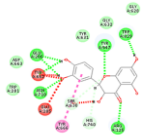	-24.371

10	4-Hydroxy-(3',4'-dihydroxyphenyl)valeric acid	Very High, 63.3	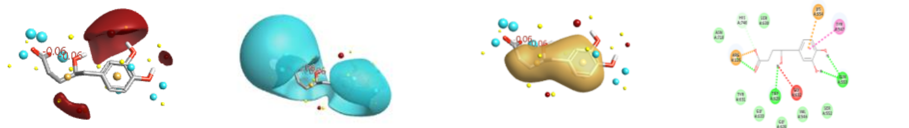	-24.335
11	5,7-Dihydroxy-8,4'-dimethoxyisoflavone	Very High, 64.2	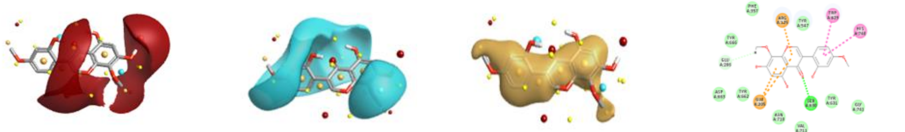	-24.232
12	Phloretin	Very High, 69.8	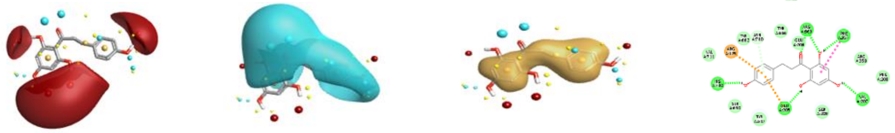	-23.174
13	5,6,7,3',4'-Pentahydroxyisoflavone	Very High, 69.9	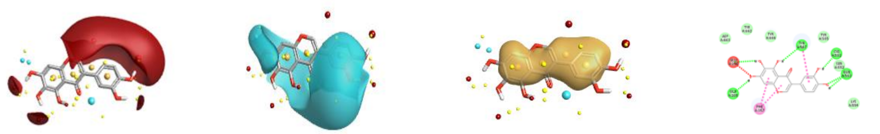	-22.787
14	Butein	Very High, 70.8	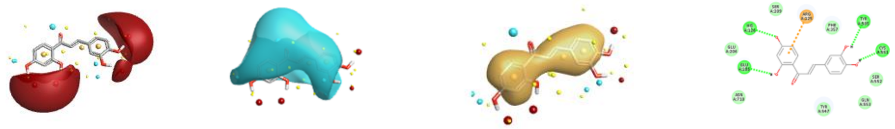	-22.776
15	5,7,8,3',4'-Pentahydroxyisoflavone	Very High, 67.0	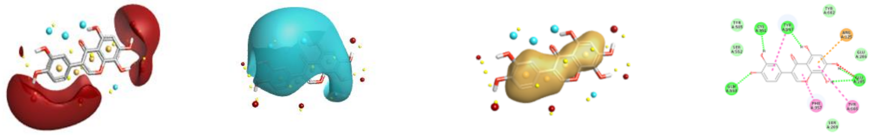	-22.776
16	Vanilloylglycine	Very High, 62.2	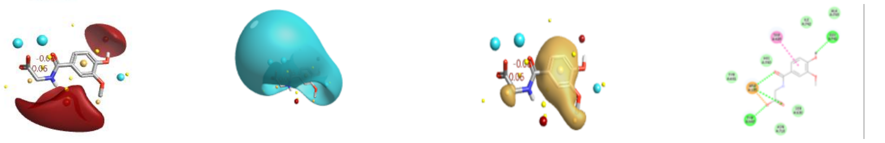	-22.665
17	7,8,3',4'-Tetrahydroxyisoflavone	Very High, 68.1	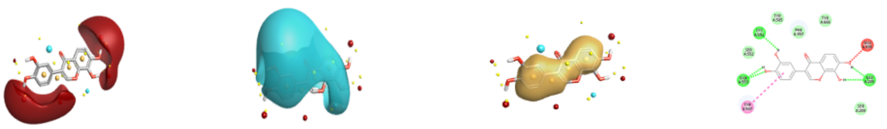	-22.655
18	Hesperetin 3'-sulfate	Very High, 68.9	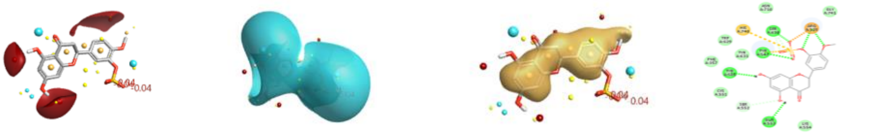	-22.539
19	Dihydroferuloylglycine	Very High, 65.7	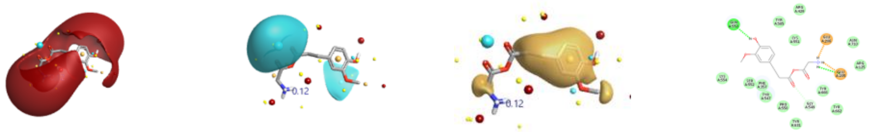	-22.474
20	trans-Resveratrol 4'-sulfate	Very High, 62.9	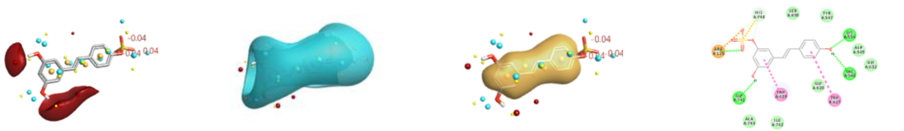	-22.355
21	Biochanin A	Very High, 63.0	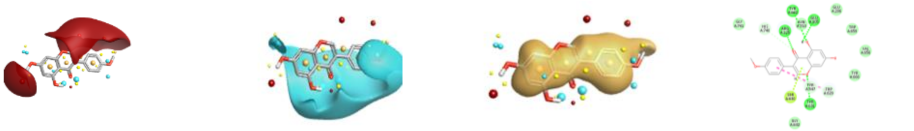	-22.306

22	Vestitone	Very High, 62.9	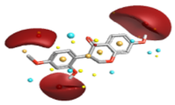	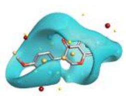	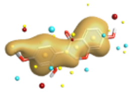	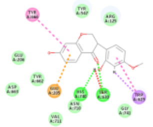	-22.18
23	m-Coumaric acid	Very High, 61.8	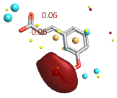	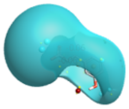	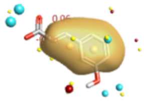	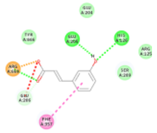	-22.101
24	Delphinidin 3-O-galactoside	Very High, 58.6	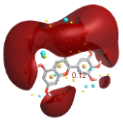	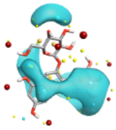	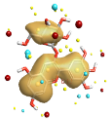	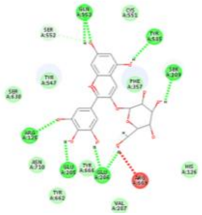	-22.073
25	Chrysin	Very High, 74.5	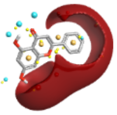	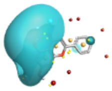	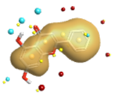	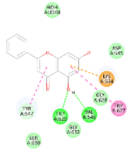	-21.4
